# Attentional capture in emotion comparison is orientation independent

**DOI:** 10.1007/s00426-022-01683-x

**Published:** 2022-05-13

**Authors:** Giulio Baldassi, Mauro Murgia, Valter Prpic, Sara Rigutti, Dražen Domijan, Tiziano Agostini, Andrea Dissegna, Carlo Fantoni

**Affiliations:** 1 Department of Life Sciences, University of Trieste, Via E. Weiss 21, 34128 Trieste, Italy; 2 De Montfort University, Leicester, UK; 3 University of Rijeka, Rijeka, Croatia

## Abstract

Recent findings on emotion comparison show a typical pattern of motor reactivity rising from attentional capture. When pairs of emotional faces are presented simultaneously, the most intense emotional face is recognized faster (Emotional Semantic Congruency—ESC effect). Furthermore, a global response speed advantage for emotional pairs with positive rather than negative average emotion intensity is observed (i.e., emotional size effect), with the choice for the happiest face resulting in a faster response than the choice for the angriest face within the pair (i.e., the happiness advantage). In two experiments, we asked whether these effects are orientation dependent, and thus linked to whether face processing is holistic or part-based. Participants were asked to choose the angriest/happiest face in emotional pairs displayed either in upright or inverted orientation and including (Experiment 1) or not including (Experiment 2) a neutral face. Beyond an overall facilitation for upright relative to inverted pairs, results showed orientation independent ESC and emotional size effects. Furthermore, the happiness advantage was present in emotional pairs of Experiment 2 but not in emotional pairs of Experiment 1, independently from face orientation. Together, results suggest that attentional capture in emotion comparison is immaterial on the type of face processing, being orientation invariant.

**Supplementary Information:**

The online version contains supplementary material available at 10.1007/s00426-022-01683-x.

## Introduction

We receive an upside-down image of the outside world through the pinholes of our eyes. Despite that, from the discovery of the blindness to local facial feature changes in upside-down faces (the Thatcher effect, Thompson, 1980), growing evidence supports the idea that the mechanisms held responsible for familiar face recognition are orientation dependent (Yin, 1969). Faces are a special object of our visual experience, they are thought to be processed holistically as wholes. Upside-down faces are more difficult to identify and discriminate than upright faces because upside down faces are part-based processed, like other non-living objects (Davidoff & Donnelly, 1990; Donnelly & Davidoff, 1999; Hochberg & Galper, 1967; McKone & Yovel, 2009; Piepers & Robbins, 2012; Tanaka & Farah, 1993; Tanaka & Sengco, 1997; Taubert et al., 2011; Valentine, 1988; Wagemans et al., 2012; Yin, 1969). This is the *face inversion effect*. Many studies showed that this effect generalizes to facial expression of emotion (Calvo & Nummenmaa, 2008; Derntl et al., 2009; Fallshore & Bartholow, 2016; Freire et al., 2000; Goren & Wilson, 2006; Jacques et al., 2007; McKelvie, 1995; Pallett & Meng, 2015; Prkachin, 2003), as well as to bodies dependent on gender (Bernard et al., 2012; Cogoni et al., 2018; Pallett & Meng, 2015). Despite this large corpus of evidence, some studies on visual search (e.g., Savage & Lipp, 2015) and emotion recognition (e.g., Aguado et al., 2009) found that inversion slows, but not alter expression recognition or effects related to it.

Our study contributes to this debated issue within the field of the face expression recognition by investigating whether the face inversion effect, which has been predominantly studied with isolated faces and emotions (Taubert et al., 2016), may affect emotion comparison. In particular, we consider a typical pattern of motor reactivity rising from attentional capture with symbolic (like numbers) and non-symbolic (like facial expressions) intensities recently discovered by Fantoni et al., (2019) and Baldassi et al., (2021) using a simultaneous comparison task. In their task with non-symbolic intensities (i.e., emotional comparison task), faces at different degrees of valence were displayed side-by-side, with one face presented to the left visual hemifield and the other one presented to the right visual hemifield. Participants were required to choose the most positive (e.g., choose the happiest) or negative (e.g., choose the angriest) face within the pair. Notably, the combination of stimuli used by the authors contrasted emotional face pairs that according to attentional capture driven by perceptual salience of facial expressions (Ferrari et al., 2008; Huang et al., 2011; Miyazawa & Iwasaki, 2010; West et al., 2009) should slow-down the choice (an intermediate emotional target face paired with a fully happy or angry face), on the one hand, vs. speed-up the choice (a fully happy or angry target face paired with an intermediate emotional face), on the other hand. In the former case, a target with low perceptual salience was coupled with a flanker with a high perceptual salience that likely behaves as an effective distractor/attractor disrupting attentional selection. In the latter case, a perceptually salient target was coupled with a rather unemotional flanker that was likely to be ignored, thus leading to fast attentional selection.

By randomizing the presentation of stimuli including/not-including a low perceptually salient (i.e., neutral) face, like half-range (i.e., a neutral face paired with a fully happy or angry face) and cross-range (i.e., an angry face paired with a happy face with equal emotional intensity) emotional pairs, Fantoni et al., (2019), but see also Baldassi et al., (2021) found the following combination of effects:

1. Response speeds increased together with absolute emotional intensity of the target face irrespective of its valence being it positive (as in the case of happy faces) or negative (as in the case of angry faces). This corresponds to a Semantic Congruency effect (Banks & Flora, 1977; Banks et al., 1975, 1976) at the level of emotion recognition, namely an Emotion Semantic Congruency effect (ESC). In particular, ESC is a general tendency for extreme, rather than intermediate emotions, to be detected more readily amongst a pair of emotions belonging to the same semantic category (i.e., angry/negative faces—amongst globally negative pairs; happy/positive faces-amongst globally positive pairs), when the comparison task requires judging the happiest/angriest. Importantly, this means that response speeds in emotion comparison result to be independent from the side of motor response (left- and right-hand) and the congruency between the spatial arrangement of the pair and any left-to-right spatial mental representation of emotional intensity. Notably, in the present paper we qualify the left-to-right spatial mental representation of emotional intensity as one consistent with findings suggesting that people automatically extract magnitude relations from faces, mentally organizing this information in a spatial format, in which the happy/positive face is represented on the right of the emotional intensity continuum relative to intermediate faces and the angry/negative face is represented on left (e.g., Holmes & Lourenco, 2011; Holmes et al., 2019; Jansari et al., 2000).
2. Global response speed advantage for globally positive (a neutral face paired with a happy face) rather than negative (a neutral face paired with an angry face) emotional pairs (i.e., emotional size effect), quantifying global emotion of a pair by means of the Average Emotion Intensity relative to the intermediate face.
3. Faster choices for the happiest rather than the angriest face within the pair (i.e., the happiness advantage).

Importantly, this combination of effects, common also to the domain of symbolic intensities as found by Baldassi et al. (2021), leads to a typical pattern of motor reactivity in which the lines connecting the speed of the target on the right and the one on the left cross over as a function of the Average Emotion Intensity in a true interaction. We named this effect “crossover effect”, as involving the speed for the angriest face within a pair to be above the speed for the happiest face at negative Average Emotion Intensity and the speed for the happiest face within a pair to be above the speed for the angriest face at positive Average Emotion Intensity. As pointed out by Fantoni et al. (2019), this crossover effect can be conceived as a typical pattern rising from attentional capture in emotion comparison and with evidence showing how emotionally salient distractors may capture exogenous attention to a significantly greater extent than neutral distractors do (Brown et al., 2020; Carretié, 2014; Ferrari et al., 2008; Micucci et al., 2020; Reeck & Egner, 2015). The crossover effect might indeed rise from extreme emotions (happy/angry) being weighed more heavily than intermediate ones in emotional pairs as they capture attention to a greater extent being perceptually salient.

## Research question and expectations

Currently, we do not know whether a similar pattern of attentional capture holds true when using an emotional comparison task with facial expressions pairs presented in inverted orientation, namely with the faces presented upside-down. It is noteworthy that this modality of face presentation should modify the way faces are processed. Given the face inversion effect, faces in upright position should be efficiently processed as wholes rather than part-based, while the reverse should apply to faces in inverted orientation. The face inversion effect should thus alter the *modalities* humans use to process facial expression of emotion, leading to an overall slowing down of motor reactivity in inverted emotional pairs (part-based processed) rather than upright (holistic-based processed) emotional pairs.

However, according to recent evidence (e.g., Aguado et al., 2009; Savage & Lipp, 2015), little is known about whether the face orientation manipulation can also alter expression recognition or effects related to it, like spatial attention involved in emotion comparison, by for instance reducing its by-products. In particular, attentional capture is known to be regulated by perceptual salience in the emotion comparison task, as leading to a precise combination of effects: namely the ESC, the emotional size effect, and the happiness advantage.

These observations lead us to the following *research question*: is the typical pattern rising from attentional capture in emotion comparison orientation dependent?

Here, we hypothesised two contrasting scenarios considering the study by West et al. (2009). To the best of our knowledge, this study was the first designed to address a similar question to the one we posit (see Huang et al., 2011 for a more recent study). Authors indeed compared the temporal order judgments for schematic faces switching from angry to neutral, and vice-versa, in upright vs. inverted orientation. Consistent with the idea that inverting a face stimulus breaks down its holistic processing and the efficient extraction of its emotional contents, authors found a prior entry effect in favour of the emotionally salient stimulus with upright but not inverted faces. If such a result generalizes to the task studied by Fantoni et al. (2019), then attentional capture in emotion comparison should not hold true for inverted facial expressions pairs, being it dependent on the type of processing. It would hold only for holistic processing instead applying to upright faces, with the crossover effect expected on the basis of attentional capture driven by perceptual salience that could not be generalized to inverted faces.

However, the transient emotional stimulation characterizing the sequences of angry/neutral faces involved in the West et al. (2009) technique, and the specific usage of schematic rather than photographic inverted faces, might not generalize to the more naturalistic case of sustained emotional stimulation characterizing the emotion comparison task of Fantoni et al. (2019). Furthermore, Fantoni et al. (2019) used photographic faces in place of schematic drawing, including both positive and negative emotions. Under these conditions, expressive qualities involving high perceptual salience might drive attentional capture more efficiently independently from the modalities in which facial expressions are perceptually processed (part-based process in inverted orientation or holistic process in upright orientation). If this occurs, fully emotional faces in both upright and inverted orientation could be prioritized by the perceptual-attentional system over other flanking/distracting stimuli competing for awareness, and the crossover effect expected on the basis of attentional capture should be generalized to both upright and inverted faces.

## Experiment 1: emotion comparison with half-range emotional pairs

**Fig. 1:**
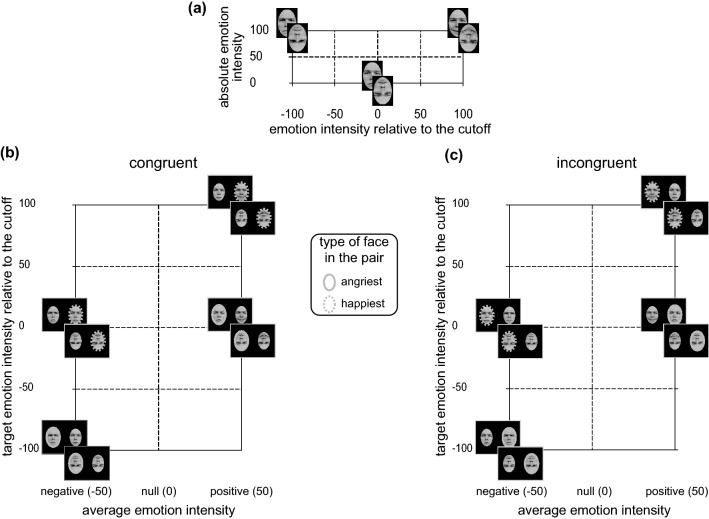
Face stimuli (**a**) and half-range emotional pairs used in Experiment 1 for the congruent (**b**) and incongruent (**c**) spatial position in both upright and inverted orientation (identity gave permission for the usage of his image but not used in our experiments). In **a**, stimuli are depicted in a Cartesian space, with the emotion intensity relative to the cutoff face along the *x*-axis and the absolute emotion intensity relative to the cutoff along the *y*-axis. On the *x*-axis the real/fully angry and the real/fully happy face define the negative and the positive extreme values of the continuum, respectively, with the neutral face defining the intermediate emotion with null intensity value (i.e., the cutoff splitting in two the emotion continuum). In **b**, **c** the half-range emotional pairs that result from the pairings of the cutoff face and the fully emotional faces shown in **a**. **b** Depicts emotional pairs in spatially congruent condition (the rightmost face in the pair is the happiest) and **c** in spatially incongruent condition (the rightmost face in the pair is the angriest). In **b**, **c** emotional pairs are represented in the average emotion intensity (*x*-axis) × target intensity relative to the cutoff (*y*-axis) Cartesian space. The type of target face in the pair is coded by the surrounding ellipses (continuous for the angriest; dashed for the happiest). Notably, the eight types of half-range emotional pairs (four congruent in b and four incongruent in **c**) in the two face orientation conditions combines into 16 half-range emotional pairs that we tested in our experiment

In order to address our *research question*, we ran an experiment using the same emotional comparison task employed by Fantoni et al., (2019) and Baldassi et al., (2021). Different from the original study, in which only facial expressions in upright orientation were used, in the present study we used facial expressions pairs with faces both in upright and in inverted orientation (see Fig. 1).

Another important difference is related the type of stimulus pairs. Indeed, in the previous studies, pairs of upright faces were randomized across the following two types: (1) half-range emotional pairs, with a real neutral face paired with a real/fully emotional face (with 100% anger or happiness in the anger-to-neutral-to-happiness in the morph continuum); and (2) cross-range emotional pairs, with an emotional face (with either 50% or 100% happiness/anger) paired with another emotional face of the same intensity, but with the opposite emotional valence (50% or 100% anger/happiness). In the present study, instead, half-range and cross-range emotional pairs were studied in two separated Experiments with both upright and inverted emotional faces to avoid doubling the duration of the original study, and to isolate the expected effect of Face Orientation from possible effects related to the type of emotional pair.

### Method

#### Participants

Forty-five participants took part in the experiment in exchange for course credits. They were Italian speakers (i.e., left-to-right reading direction), naïve to the purpose of the study, and had normal/corrected-to-normal visual acuity. Data from 3 participants were excluded from the analysis because of technical problems during data collection. Consequently, data from 42 participants (35 females: average age = 19.71 ± 2.12 SD; age range = [18–32]) were analysed. We conducted a sensitivity analysis with G-Power 3.1 (Faul et al., 2007) on our sample size with *α* err. prob. = 0.05, power (1 − *β* err. prob.) = 0.8 in order to establish the minimal detectable effects resulting from our experimental design. These resulted to be in the medium-to-large range with a critical *F* = 2.05 and a *η*_*p*_^2^ = 0.17 and a critical two-tailed *t* of about 2.03 05 and a *d* = 0.50.

Participant’s handedness was measured with the 10-item Edinburgh Handedness Inventory (Oldfield, 1971), which revealed an average of 55.48 (SD =  ± 49.14; min. to max. range = [− 70 to 100]). Participants were randomly assigned to one of the two conditions of instruction order (A or B). The participants assigned to the order A completed the experiment following the instruction “choose the angriest face of the two” in the first block and the instruction “choose the happiest face of the two” in the second block; the participants assigned to the order B did the opposite. Twenty participants completed the experiment in the order A, and 22 completed it in the order B.

#### Apparatus and stimuli

Stimulus presentation and response recording were controlled by a custom-made E-Prime 2.0 program. Facial expressions pairs were presented on a 22″ Dell P2214H monitor with 1920 × 1080 pixels resolution via PC, in a dimly lit laboratory with the participant comfortably seated facing the screen at an average distance of 38 cm. A QWERTY keyboard was used for collecting responses and it was positioned on the desk between the participant and the monitor. In order to ensure a comfortable posture, the distance between the participant and the keyboard was adapted to the participant arm length, with only the “d” and “j” keys (keys’ distance = 8 cm) activated during the experiment and centred along the participants’ sagittal axis.

We used two different sets of facial expressions of emotions in order to create our emotional pairs as follows: line-drawn faces in the training session and coloured photographs in the experimental session. All facial expressions pairs consisted of two faces as follows: both displayed either in upright or in inverted orientation, centred on the horizontal axis of the screen (i.e., the midline of the two faces corresponding to the vertical midline of the screen), with a centre-to-centre (i.e., nose-to-nose) distance equal to 19.6° (at the average viewing distance of 38 cm). The set of faces in upright orientation was the same of the set of faces in inverted orientation, but with faces rotated 180°. Each face was masked by an oval vignette hiding hair and ears, was presented on a black surround, and had a horizontal × vertical extent of 12.0° × 16.8°.

In the training session we used a set of six black and light grey drawn facial stimuli (three in upright and three in inverted orientation) with a single unisexual model reproducing an angry, a neutral or a happy face (created according to an on-line tutorial, see Fantoni et al., 2019 for details). We used eight facial expressions pairs resulting from the combination of 2 types of stimuli (angry-neutral, neutral-happy) × 2 spatial Congruency with the left/right mental representation of valence of emotion (congruent—with the happy/positive face on the right, incongruent − with the angry/negative face on the right) × 2 face orientation (inverted, upright). The training session lasted 16 trials, with the full random presentation of the 8 facial expressions pairs repeated 2 times.

In the experimental session we used eight Caucasian Characters (four females and four males) selected from the Radboud University Nijmegen set (Langner et al., 2010, Character numbers: 1, 2, 4, 19, 20, 30, 46, and 71, see Fantoni et al., 2019 for details). As depicted in Fig. 1a, for each character we obtained a set of six facial stimuli (i.e., three in upright and three in inverted orientation), belonging to the anger-to-neutral-to-happiness continuum, with four facial expressions displaying basic emotions (i.e., real and full facial expression of emotion in upright/inverted orientation) and two real neutral facial expressions (upright/inverted). As depicted in Fig. 1b, c, the neutral face of each Character was paired with the fully emotional faces in order to obtain eight types of half-range emotional pairs resulting from the combination of 2 face orientation × 2 spatial congruency × 2 average emotion intensity relative to the cut-off (− 50, 50).

### Experimental design

Our eight types of half-range emotional pairs, when combined with the two conditions of Response Side resulting from the manipulation of the type of instruction (“choose the happiest”, “choose the angriest”) defined our 2 × 2 × 2 × 2 cross-over experimental design. Our experimental design thus consisted of 16 conditions resulting from the factorial combination of four within-subject factors: 2 face orientation (inverted, upright) × 2 response side (left, right) × 2 spatial congruency (congruent − with the happy/positive face on the right, incongruent − with the angry/negative face on the right) × 2 average emotion intensity (− 50, 50). Instruction ordering with “choose the angriest” first or “choose the happiest” first was treated as a balancing variable. In sum, each experimental session comprised a total of 64 trials resulting from the factorial combination of 8 characters (treated as repetitions) × 2 average emotion intensity × 2 spatial congruency × 2 face orientation.

#### Procedure

The procedure resembled the one used in Fantoni et al., (2019) and Baldassi et al., (2021). It included a sequence of the following six events: (1) Edinburgh Handedness Inventory; (2) general oral instructions about the experiment; (3) a first training session introduced by on-screen instruction (“choose the angriest/happiest”, depending on Instruction Ordering); (4) a first experimental session introduced by the same on-screen instruction of the first training session; (5) a second training session introduced by on-screen instruction which was different from the instruction of the first training session; (6) a second experimental session introduced by the same on-screen instruction of the second training session. On-screen instructions informed participants that they have to choose among a pair of horizontally aligned faces which of the two appear to be the angriest/happiest, using the keys on keyboard with the corresponding spatial position (“d” press if target on the left vs. “j” press if target on the right). The experiment was composed of a total of 32 training trials and 128 experimental trials. The trial temporal structure was the same as in Fantoni et al. (2019) with a fixation screen (a white cross on a black background) lasting about 2000 ms, followed by a blank screen lasting 200 ms, and in turn by a stimulus screen which was self-terminated by the participant response (minimum to a maximum duration, 190–2890 ms, respectively). A blank masking screen lasting about 3000 ms terminated the trial after participant response.

#### Data analysis

We applied the exact same exclusion criteria used in previous studies of our group (Baldassi et al., 2021; Fantoni et al., 2019) for the selection of valid responses in order to make our results comparable and generalizable. From a total collection of 5362 responses, we excluded the following: (1) 221 incorrect responses (e.g., the choice of the angriest face when the instruction required to “choose the happiest face in the pair” or vice-versa); (2) 7 correct responses falling outside the [200 ms, 2500 ms] response time, RT, limit; (3) 96 correct responses falling outside ± 3 SD from the predicted value of the best *linear mixed effect* model, *lme*, with all experimental factors and their interactions as fixed structure (average emotion intensity, response side, spatial congruency, and face orientation), and participants as random intercepts. We transformed the remaining 5038 values of RT into values of response speeds (1000/RT). Such a transformation was motivated by the homology between actual speed and response accuracy and by its capacity to normalize the skewed distribution of RTs increasing statistical power and reducing the likelihood of spurious outlier removal (Miller, 1991; Ratcliff, 1993; Whelan, 2008).

Considering the 16 experimental conditions of our experimental design, the average number of valid trials per condition was equal to the following: 14.99 ± 1.30 SD, range = [9, 16], corresponding to an average accuracy of about 0.94 ± 0.06 SD and an average *z* score of proportion of correct responses of 1.22 ± 0.42 SD. Average *z* score of proportion were calculated keeping the ratio between the deviations of the individual proportion from the hypothesized value of population proportion in the null hypothesis, *p*_*0*_ = 0.75, with a guess rate = 0.5, and a SD of the sampling distribution, *σ* = 0.153.

Individual values of response speeds were used to extract for each participant four individual synthetic index of happiness advantage: one for each condition resulting from the combination of two face orientation × 2 spatial congruency conditions. Each individual index of happiness advantage was calculated subtracting the individual value of the best fitting *lme* regressor’s intercept for the selection of the angriest faces calculated across two pairs from the individual value of the best fitting *lme* regressor’s intercept for the selection of the happiest faces, in both upright and inverted Face orientation conditions. In particular, for spatially congruent pairs these regressors correspond to the line connecting the average response speed of a left-hand response to a fully angry face flanked by a neutral face and the line connecting the average response speed of a left-hand response to a neutral face flanked by a fully happy face. For spatially incongruent pairs they correspond to the line connecting the average response speed of a right-hand response to a fully angry face flanked by a neutral face and the line connecting the average response speed of a right-hand response to a neutral face flanked by a fully happy face. Happiness advantage’ indices synthetically quantify how much the motor reactivity is biased by the selection of the most positive vs. negative face within the pair, with positive values indicating an imbalance in favour of the selection of the most positive face (when the average emotion intensity is null), depending on the spatial congruency of the pair and on the face orientation.

We selected for all our *lme* models the maximal random effects structure justified by our experimental design (Barr et al., 2013). Our models involved by-subject random intercepts and slopes, with our balancing variable (the instruction ordering) used as an additional random intercept. We selected the fixed structure of our *lme* models according to a stepwise procedure contrasting *lmes* of increasing complexity depending on the number of fixed factors, modelled by the following factors of our experimental design: average emotion intensity, spatial congruency, response side, and orientation. handedness was categorized according to median split (i.e., small vs. large) and used as a covariate in our preliminary *lme* analyses. Consistently with the results of Fantoni et al., (2019) and Baldassi et al., (2021), preliminary *lme* analyses revealed no reliable interaction between accuracy, handedness, speeds, and other experimental factors. A *lme* model including a reliable speed–accuracy positive correlation, *F*(1, 343.75) = 74.95, *p* < 0.001, *η*_*p*_^2^ = 0.118, 95% CI [0.061, 0.146], with accuracy increasing of about 0.11 ± 0.01 per cent every unit increment of speed, *t*(343.75) = 8.66, *p* < 0.001, *d* = 0.93, indeed achieve a larger goodness of fit than a model combining Handedness with all other factors of our experimental design, *χ*^2^(68) = 110.12, *p* < 0.001, AIC(6) = − 1296.8, vs. AIC(68) = − 1282.9, BIC(6) = − 1269.76, vs. BIC(68) = − 976.24. Furthermore, we found no reliable interaction and main effects linked to the Gender of our face stimuli with other experimental factors as demonstrated by the higher goodness of fit of an *lme* model not including the gender [AIC(23) = 1563.2, vs. AIC(39) = 1601.3, BIC(23) = 1713.3, vs. BIC(39) = 1855.8]. This demonstrated the set of our face stimuli was well balanced in terms of perceptual-based gender features that according to Pallett and Meng (2015) in some cases might covariate with emotion recognition. Due to these preliminary results, we decided to focus the main analyses on individual response speeds, the happiness advantage indices and our main experimental factors as fixed and random effects for our *lme* statistic.

As statistical inferential measures we provided the following: (1) type III-like two-tailed *p* values for significance estimates of *lme*’s fixed effects and parameters adjusting for the *F* tests the denominator degrees-of freedom with the Satterthwaite approximation; (2) estimates of the *lme* goodness of fit based on AIC-index, BIC-index, and *χ*^2^; (3) estimates of effect size based on the concordance correlation coefficient *r*_c_, partial eta squared *η*_*p*_^2^ (for the interactions and main effects of the F-tests), and Cohen’s *d* (for the post-hoc analyses performed on *lme* estimated coefficients with paired two sample *t* tests with unequal variance).

### Results

**Fig. 2:**
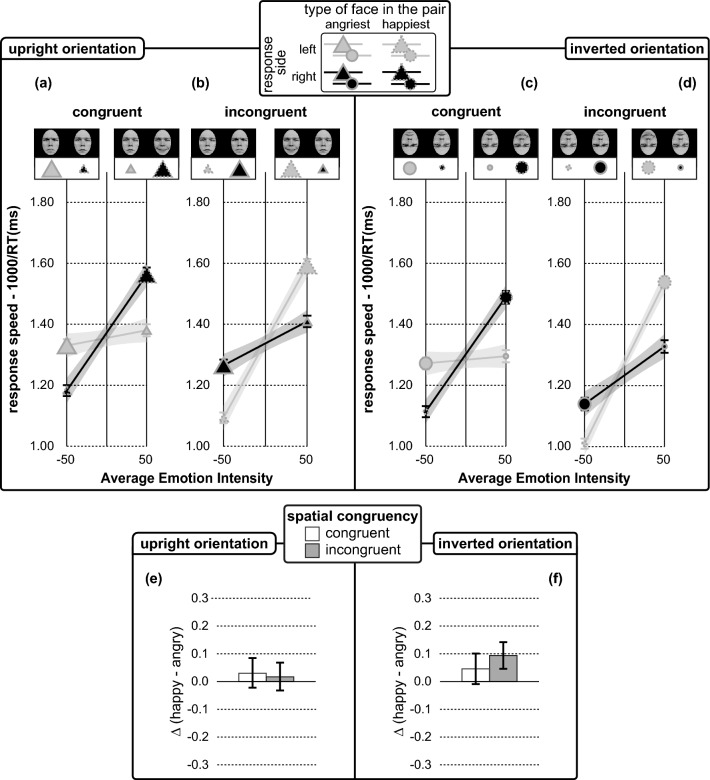
Emotion comparison performance of Experiment 1 indexed by response speeds (in panels from **a** to **d**) and happiness advantages (in panels **e**, **d**). Error bars represent ± 1 Standard error of the mean, SEM. **a**–**d** Average response speeds in spatially congruent (**a**, **c**) and spatially incongruent (**b**, **d**) conditions, as a function of average emotion intensity (*x*-axis), with the shape of the symbols encoding for (see the legends on top) the face orientation condition (upright—triangles in panels **a**, **b**; inverted circles in panels **c**, **d**): the colour filling the symbols encoding for the response side (mid grey for left; black for right), and the outline of the symbols encoding the type of target face (continuous for angriest; dashed for happiest). The size of the symbols represents the absolute emotion intensity (small for neutral; large for fully angry/happy emotional face). Mid grey and black lines are the *lme* model regression lines for left/right response side conditions, with the shaded bands corresponding to ± 1 SEM of the regression. Panels **e**, **f** depict average happiness advantage for spatial congruent and incongruent conditions as encoded by the colour of the bar (see the legends on top)

Figure 2 illustrates the patterns of average values of response speeds (Fig. 2a–d) and happiness advantage (Fig. 2e, f) for half-range emotional pairs.

We consider a *lme* model on individual values of response speeds including average emotion intensity, face orientation, response side, and spatial congruency as fixed effects, *r*_c_ = 0.72, 95% CI [0.71, 0.73]. This revealed an average emotion intensity × response side × spatial congruency interaction, *F*(1, 284.90) = 310.14, *p* < 0.001, *η*_*p*_^2^ = 0.521, 95% CI [0.445, 0.583], consistent with an orientation invariant ESC effect. The ESC effect indeed held true for facial expressions pairs displayed in both upright and inverted face orientation. In upright face orientation presented in Spatially Congruent condition (Fig. 2a, b), the selection of the happiest target on the right was faster than the selection of the angriest target on the left for positive average emotion intensity (*M*_happiest/emotional_ = 1.56 ± 0.05, vs. *M*_angriest/neutral_ = 1.38 ± 0.05,, *t*(40.21) = 4.72, *p* < 0.001, *d* = 1.49) and vice-versa for negative average emotion intensity [*M*_angriest/emotional_ = 1.32 ± 0.04, vs. *M*_happiest/neutral_ = 1.18 ± 0.04, *t*(41.44) = 5.09, *p* < 0.001, *d* = 1.58]. The reverse pattern was observed in spatially incongruent condition, with the selection of the happiest target on the left being faster than the selection of the angriest target on the right for positive average emotion intensity [*M*_happiest/emotional_ = 1.60 ± 0.04, vs. *M*_angriest/neutral_ = 1.41 ± 0.04, *t*(40.85) = 5.37, *p* < 0.001, *d* = 1.68], and vice-versa for negative average emotion intensity [*M*_angriest/emotional_ = 1.27 ± 0.04, vs. *M*_happiest/neutral_ = 1.10 ± 0.04, *t*(41.35) = 6.64, *p* < 0.001, *d* = 2.07]. In inverted face orientation (Fig. 2c, d), we found similar results, both in (1) spatially congruent condition with positive [*M*_happiest/emotional_ = 1.49 ± 0.04, vs. *M*_*angriest*/neutral_ = 1.30 ± 0.04, *t*(40.63) = 5.32, *p* < 0.001, *d* = 1.67] and negative [*M*_angriest/emotional_ = 1.27 ± 0.03, vs. *M*_happiest/neutral_ = 1.11 ± 0.03, *t*(40.98) = 5.20, *p* < 0.001, *d* = 1.62] average emotion intensity, and (2) spatially incongruent condition with positive [*M*_happiest/emotional_ = 1.53 ± 0.04, vs. *M*_angriest/neutral_ = 1.31 ± 0.04, *t*(41.03) = 6.24, *p* < 0.001, *d* = 1.95] and negative [*M*_angriest/emotional_ = 1.14 ± 0.04, vs. *M*_happiest/neutral_ = 1.01 ± 0.03, *t*(40.22) = 4.60, *p* < 0.001, *d* = 1.45] average emotion intensity.

As regards the emotional size effect, the *lme* analysis revealed a main effect of average emotion intensity, *F*(1, 36.21) = 195.26, *p* < 0.001, *η*_*p*_^2^ = 0.844, 95% CI [0.731, 0.891]. response speeds increased steadily as average emotion intensity grew larger, *β* = 0.0027 ± 0.0002, *t*(41.47) = 14.37, *p* < 0.001, *d* = 4.46, from negative to positive average emotion intensity.

As consistent with the face inversion effect, the *lme* analysis revealed a main effect of face orientation, *F*(1, 284.62) = 55.77, *p* < 0.001, *η*_*p*_^2^ = 0.164, 95% CI [0.093, 0.240], with faster responses for half-range emotional pairs in upright over inverted orientation (Fig. 2, triangles vs. circles), *M*_Upright_ = 1.24 ± 0.04, vs. *M*_Inverted_ = 1.13 ± 0.03, *t*(293.93) = 7.52, *p* < 0.001, *d* = 0.88.

Finally, the *lme* analysis revealed a pattern of effects that is not fully consistent with the typical pattern of responses rising from attentional capture in emotion comparison: a significant main effect of spatial congruency, *F*(1, 284.80) = 5.42, *p* < 0.05, *η*_*p*_^2^ = 0.019, 95% CI [0.000, 0.060] (with *M*_Incongruent_ = 1.23 ± 0.03, vs. *M*_Congruent_ = 1.17 ± 0.03, *t*(291.69) = 3.08, *p* < 0.01, *d* = 0.36) and its interaction with average emotion intensity, *F*(1, 284.92) = 40.54, *p* < 0.001, *η*_*p*_^2^ = 0.124, 95% CI [0.061, 0.197] (*lme* estimated gain from negative to positive Average Emotion Intensity of about 0.21 ± 0.03 for spatially congruent emotional pairs, and an additional *lme* estimated gain for spatially incongruent emotional pairs of about 0.13 ± 0.04, *t*(168.9) = 3.27, *p* < 0.001, *d* = 0.50).

No other main effects or interaction were observed (*p*s > 0.06). Notably, the spatial congruency × response side interaction diagnostic of a happiness advantage was not significant, *F*(1, 284.79) = 3.61, *p* = 0.06. The speed of the choice for positive (*lme* estimated average speed for incongruent/left and congruent/right conditions, *M*_happiest_ = 1.13 ± 0.03) and negative (*lme* estimated average speed for congruent/left and incongruent/right conditions, *M*_angriest_ = 1.13 ± 0.03) emotions *t*(293.49) = 0.47, *p* = 0.60 was indeed similar.

As a final *lme* analysis we better address this unexpected lack of spatial congruency × response side interaction analysing the individual values of happiness advantage, with a *lme* model including face orientation and spatial congruency as fixed factors. The results of the analysis were confirmatory. They indeed revealed no significant interactions or main effects, *r*_c_ = 0.58, 95% CI [0.50, 0.63]. We then contrasted individual values of happiness advantage against the reference null value representing a full balance between responses given to the happiest and the angriest faces: no significant differences were found for both the subset of half-range emotional pairs in upright, *M*_values of happiness advantage_ = 0.006 ± 0.012, vs. 0, *t*(41) = 0.51, *p* = 0.62, *d* = 0.15, and inverted, *M*_values of happiness advantage_ = 0.034 ± 0.022, vs. 0, *t*(41) = 1.53, *p* = 0.13, *d* = 0.50, face orientation. Such results corroborated the absence of the happiness advantage with the set of half-range emotional pairs studied in the current experiment: namely, emotional pairs including a real neutral face in both inverted and upright orientation.

### Discussion

Overall, the results are consistent with an orientation independent emotional capture effect evoked by our emotion comparison task, as corroborated by the following three theoretically relevant effects revealed by the *lme* analysis: (1) a 3-way interaction between average emotion intensity × response side × spatial congruency, consistent with ESC; (2) a main effect of average emotion intensity, consistent with size effect; (3) a main effect of face orientation, consistent with a face inversion effect.

As regards the ESC, participants’ motor reactivity increased when targets expressed the strongest emotions, independently from the response side and the spatial congruency. This pattern of motor reactivity resulted in a full crossover, with the speed for the angriest face within a pair above the speed for the happiest face at negative average emotion intensity, and vice-versa at positive average emotion intensity. This crossover was fully reversed in terms of response side in the two spatial congruency conditions. Importantly, the crossover effect was invariant over face orientation conditions. A similar invariance characterized the emotional size effect, with motor reactivities being larger for globally positive rather than globally negative emotional pairs in both upright and inverted face conditions. Finally, we found an overall slowing down of motor responses in the inverted face condition. This main effect of face orientation was consistent with a general face inversion effect (Yin, 1969). This suggests a holistic processing of facial expressions of emotion in our emotion comparison task. However, given that such holistic processing did not alter the crossover pattern in our task, we can conclude that attentional capture is independent of Face Orientation and of the type of face processing.

As regards the pattern of effects that were not expected based on the typical pattern of responses rising from attentional capture in emotions comparison (the main effect of spatial congruency and its interaction with average emotion intensity) this was both statistically and theoretically uncertain. The direction of the effect of spatial congruency was indeed inconsistent with any interpretation including the congruency with the mental spatial representation of emotion as a determinant factor for priming spatial attention. We indeed found that responses were globally faster for emotional pairs in spatially incongruent over congruent position. Furthermore, such effect was modulated by the average emotion intensity, with the difference between the motor reactivity in globally positive and globally negative emotional pairs being larger for congruent than incongruent conditions. Both effects should be interpreted with caution being their size well below the estimated minimum detectable effect in our experimental design.

Importantly, our results differently from previous findings of Fantoni et al., (2019) and Baldassi et al., (2021) in which half-range and cross-range emotional pairs were randomized, did not reveal any spatial congruency × response side interaction diagnostic of a happiness advantage. Such a lack of interaction provided us the motivation for realizing Experiment 2.

## Experiment 2: emotion comparison with cross-range emotional pairs

In Experiment 1, differently from Fantoni et al. (2019), we did not find a reliable happiness advantage. This suggests that when the emotion comparison task includes only half-range emotional pairs, and not cross-range emotional pairs, the motor reactivity is globally balanced over response sides. A possible explanation for this result might be the different types of stimulus pairs we used compared to the original study (Fantoni et al., 2019). When the emotional intensity of two faces within a pair is similar, although with opposite valence (as in cross range pairs), the attentional system is faced with a conflict, that it might solve prioritizing the most perceptually salient expression: i.e., the happiest faces as expected with realistic faces as those used in the current experiments (Becker et al., 2011, 2012; Fantoni & Gerbino, 2014; Fantoni et al., 2016). It is likely that such a conflict does not occur in half-range emotional pairs, as observed in Experiment 1, in which the discrepancy between emotion intensity of the faces within a pair was large and thus the attentional system prioritized the most intense emotional expression, as consistent with an effect of attentional capture driven by perceptual salience.

**Fig. 3:**
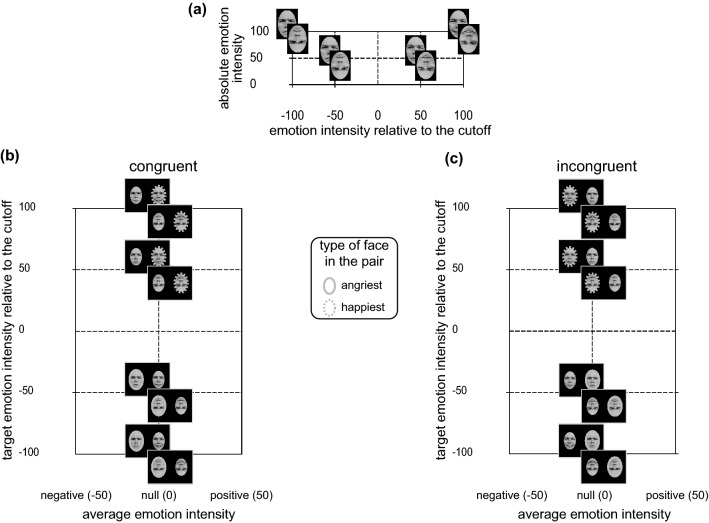
Face stimuli (**a**) and cross-range emotional pairs used in Experiment 2 for the congruent (**b**) and incongruent (**c**) spatial position in both upright and inverted orientation (identity gave permission for the usage of his image but not used in our experiments). See caption of Fig. 1 for further explanation

In Experiment 2, we used a set of emotional pairs that—differently from those of Experiment 1—have a null average emotion intensity. This included, in each pair, both the angry/negative and the happy/positive face, namely cross-range emotional pairs. Our aim was to further explore the occurrence of the happiness advantage and its dependence on face orientation. Notably, we kept constant the number of trials as the one used in Experiment 1 studying only cross-range emotional pairs characterized by emotional faces with opposite valence (one happy and one angry) with either intermediate of fully absolute emotion intensity (50 or 100), in both upright and inverted orientation (Fig. 3). Differently from Experiment 1, our pairs thus differed in term of their target absolute emotion intensity but not in term of their average emotion intensity (resulting always null in our cross-range pairs).

Beyond the expected face inversion effect observed in Experiment 1, the experimental design of Experiment 2 allowed us to test for a further effect: namely an emotional distance effect, which was originally found by Fantoni et al., (2019) and Baldassi et al., (2021). According to the emotional distance effect, response speeds should increase as the absolute difference between the emotions expressed by the two faces within the pair increases. Faster responses are expected for cross-range emotional pairs displaying fully rather than intermediate emotion expression, as the absolute emotional distance along the valence continuum between a fully (100) happy and a fully (− 100) angry face equals 200 which is two times the absolute distance between an intermediate (50) happy and an intermediate (− 50) angry face which is equal to 100.

### Method

#### Participants

Data from 46 participants (different from those of Experiment 1; 36 females and 10 males; average age = 20.91 ± 6.10 SD; age range = [18–49]; mean Edinburgh Handedness Inventory = 63.35, SD =  ± 36.05; min. to max. range = [− 47 − 100]) were analysed in Experiment 2. The same sensitivity analysis conducted in Experiment 1 but including a sample size = 46 established similar medium-to-large minimal detectable effects with a critical *F* = 4.17, *η*_*p*_^2^ = 0.15 and a critical two-tailed *t* of about 2.01. Participants were randomly assigned to one of the two conditions of instruction order (A or B, see Participant subsection of Experiment 1). Twenty-five participants completed the experiment in the order A, and 21 completed it in the order B.

#### Apparatus and stimuli

The apparatus was the same as in Experiment 1. As in Experiment 1, we used two different sets of facial expressions of emotions in order to compose our emotional pairs: line-drawn faces in the training session and coloured photographs in the experimental session.

Cross-range emotional pairs of Experiment 2 had the same geometrical proprieties of half-range emotional pairs of Experiment 1. In the training session, we used four emotional pairs (pairing an angry and a happy face) resulting from the combination of 2 spatial congruency × 2 face orientation. The training session lasted 16 trials (four emotional pairs × four repetitions).

In the experimental session we used a set of emotional faces extracted from the same set of characters used in Experiment 1. As depicted in Fig. 3a, for each character we obtained a set of eight face stimuli (i.e., four in upright and four in inverted orientation), belonging to the anger-to-neutral-to-happiness continuum: four fully emotional faces displaying basic emotions (the *real* facial expression of anger/happiness in upright/inverted orientation—these were the same used in Experiment 1), four morphed facial expressions displaying intermediate emotions (a mixture of 50% anger/happiness and 50% neutral in upright/inverted orientation). For a detailed description of morphing technique see Fantoni et al., (2016). As depicted in Fig. 3b, c, the faces with opposite emotion intensity relative to the cut-off expression but equal intensity of each character were paired in order to obtain the eight types of cross-range emotional pairs resulting from the combination of 2 face orientation × 2 spatial congruency × 2 target absolute emotion intensity (50, 100). Notably, differently from Experiment 1 in which we tested half-range emotional pairs with non-null average emotional intensity but constant absolute emotional distance along the valence continuum between the fully emotional and the neutral face (i.e., 100), in Experiment 2 we tested cross-range emotional pairs with null Average Emotional Intensity, though variable absolute emotional distance along the valence continuum between the faces within the pair: 100 for pairs with a target absolute emotion intensity equal to 50, and 200 for pairs with a target absolute emotion intensity equal to 100. Such a difference is appreciable in panels 3b and 3c in which our 16 types of cross-range emotional pairs cut in two the common Cartesian space used to represent the half-range emotional pairs of Experiment 1 now all laying along the vertical line through the origin.

#### Experimental design

Our eight types of cross-range emotional pairs when combined with our two conditions of response side defined a 2 × 2 × 2 × 2 cross-over design as resulting from the combination of 2 face orientation × 2 response side × 2 spatial congruency × 2 target absolute emotion intensity, with instruction ordering used (as in Experiment 1) as a balancing variable. Our experimental design consisted of 16 conditions resulting from the factorial combination of 4 within subjects’ factors: 2 face orientation (inverted, upright) × 2 response side (left, right) × 2 spatial congruency (congruent—with the happy/positive face on the right, incongruent—with the angry/negative face on the right) × 2 target absolute emotion intensity (50, 100). Each experimental session comprised a total of 64 trials resulting from the factorial combination of 8 characters × 2 target absolute emotion intensity × 2 spatial congruency × 2 face orientation.

#### Procedure

The procedure was the same as in Experiment 1.

#### Data analysis

From a total collection of 5877 responses, we applied the same exclusion criteria used in Experiment 1 and excluded (1) 129 incorrect responses, (2) 6 correct responses falling outside the [200 ms, 2500 ms] RT limit, and (3) 106 correct responses falling outside ± 3 SD from the predicted value of the best generalized *lme* regression model. We similarly transformed the remaining 5636 individual values of RT into individual values of response speeds (i.e., 1000/RT). The average number of valid trials per experimental condition resulted to be equal to the following: 15.32 ± 1.11 SD range = [9, 16] (corresponding to a global average accuracy of about 0.96 ± 0.05 SD and an average *z* score of proportion of correct responses of 1.35 ± 0.36 SD).

We analysed the same indices of emotion comparison performance using the same *lme* analyses based on the maximal random effects structure justified by our experimental design and used the same estimates of significance, of the goodness of fit and of effect size of Experiment 1. As in Experiment 1, individual values of response speeds were used to extract, for each participant, eight individual synthetic index of happiness advantage: 4 for the target absolute intensity condition equal to 50 (resulting from the combination of 2 face orientation × 2 spatial congruency) and 4 for the target absolute intensity condition equal to 100. Each individual index of happiness advantage was calculated so to be fully homologous to the value extrapolated in Experiment 1 (from the intercept of the best fitting *lme* regressors of average responses to half-range emotional pairs with non-null average emotional intensity). In the case of cross-range emotional pair, however, given that the average emotional intensity was null no such extrapolation was needed, and individual index of happiness advantage was directly calculated on the basis of actual average response speeds. In particular, we subtracted the individual average response speed for the selection of the angriest and the happiest face within the same pair, in both upright and inverted face orientation conditions for pairs displaying both full emotional (target absolute intensity = 100) and intermediate emotions (target absolute intensity = 50). Notably, only the happiness advantage’ indices calculated on cross-range emotional pair with the intermediate emotions were fully comparable to those extrapolated from responses to half-range emotional pairs studied in Experiment 1, as these two types of emotional pairs were equal in terms of absolute emotional distance along the valence continuum (|− 50| +|50| =|0| +|± 100|).

As in Experiment 1, our preliminary analysis on the speed accuracy correlation and on the effect of handedness justify the rationale of focusing the main analyses on individual response speeds and happiness advantages. In particular, a *lme* model including a reliable speed–accuracy positive correlation, *F*(1, 285.99) = 95.04, *p* < 0.001, *η*_*p*_^2^ = 0.120, 95% CI [0.073, 0.160], with accuracy increasing of about 0.09 ± 0.01 per cent every unit increment of speed, *t*(286) = 9,75, *p* < 0.001, *d* = 1.15, indeed achieve a larger goodness of fit than a model combining Handedness with all other factors of our experimental design, *χ*^2^(30) = 34.90, *p* = 0.25, AIC(6) = − 1621.6, vs. AIC(36) = − 1596.5, BIC(6) = − 1594, vs. BIC(36) = − 1430.8. Last, we found no reliable interaction and main effects linked to the gender of our face stimuli with other experimental factors as demonstrated by the higher goodness of fit of an *lme* model not including the gender [AIC(23) = 3713.5, vs. AIC(39) = 3877.4, BIC(23) = 3866.1, vs. BIC(39) = 4136.3].

### Results

**Fig. 4:**
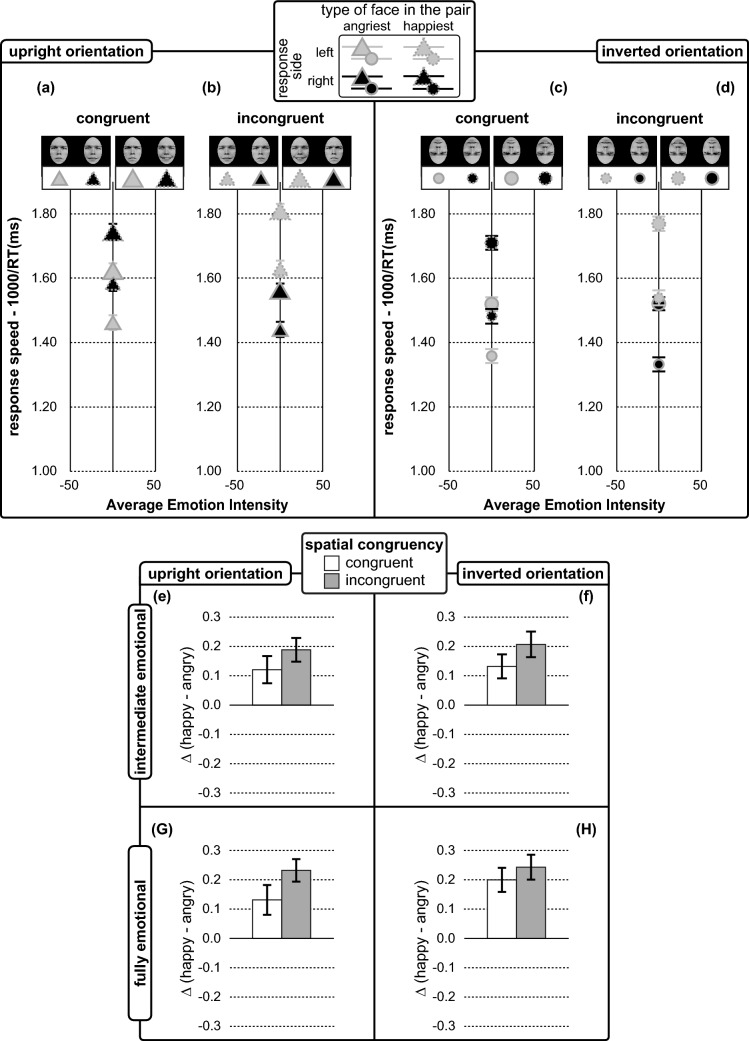
Emotion comparison performance of Experiment 2 indexed by response speeds (in panels from **a**–**d**) and happiness advantages (in panels **e**–**h**). Error bars represent ± 1 SEM See caption of Fig. 2 for further explanation on variable encoding and legends

The *lme* analysis on individual values of response speeds and happiness advantages obtained in Experiment 2 was consistent with our expectations. In particular, as depicted in Fig. 4 (same rationale and variable encoding used in Fig. 2), the pattern of motor reactivity supported the occurrence of the face inversion effect (triangles in Fig. 4a, b globally higher than circles in Fig. 4d), happiness advantage (Fig. 4e–h) and the emotional distance effect (smaller symbols globally lower than larger symbols in Fig. 4a–d).

The *lme* analysis on individual values of response speeds including target absolute emotion intensity, face orientation, response side, and spatial congruency as fixed effects, *r*_c_ = 0.70, 95% CI [0.69, 0.71], see Fig. 4 (same rationale and variable encoding used in Fig. 2), revealed the following three significant effects: first, a face inversion effect revealed by a main effect of face orientation, *F*(1, 208.93) = 13.52, *p* < 0.001, *η*_*p*_^2^ = 0.061, 95% CI [0.013, 0.132], with faster *lme* estimated responses for cross-range emotional pairs in upright over inverted orientation, *M*_Upright_ = 1.33 ± 0.03, vs. *M*_Inverted_ = 1.25 ± 0.03, *t*(277.95) = 3.79, *p* < 0.001, *d* = 0.45. Second, an emotional distance effect signalled by the main effect of target absolute emotional intensity, *F*(1, 98.33) = 304.03, *p* < 0.001, *η*_*p*_^2^ = 0.756, 95% CI [0.671, 0.808]. In particular, the speed of judgements increased with the target absolute emotion intensity both for the “choose the angriest” task, *M*_Target Absolute Emotional Intensity = 50_ = 1.39 ± 0.04, vs. *M*_Target Absolute Emotional Intensity = 100_ = 1.55 ± 0.04, *t*(154.10) = 13.32, *p* < 0.001, *d* = 2.14, and “choose the happiest” task, *M*_Target Absolute Emotional Intensity = 50_ = 1.54 ± 0.10, vs. *M*_Target Absolute Emotional Intensity = 100_ = 1.75 ± 0.09, *t*(24.17) = 12.87, *p* < 0.001, *d* = 4.94. Third, a happiness advantage, not observed on half-range emotional pairs of Experiment 1, revealed by the significant response side × spatial congruency interaction, *F*(1, 208.95) = 10.24, *p* < 0.01, *η*_*p*_^2^ = 0.047, 95% CI [0.007, 0.113]. Response was faster for the selection of the happiest target within a pair (with *lme* estimated average speed *M* = 1.39 ± 0.03, resulting by pooling responses on target faces in incongruent/left *M* = 1.68 ± 0.04 and congruent/right *M* = 1.62 ± 0.04 conditions) over the angriest target within the pair (with *lme* estimated average speed *M* = 1.20 ± 0.02, *t*(311.76) = 10.73, *p* < 0.001, *d* = 1.21, resulting by pooling responses on congruent/left *M* = 1.48 ± 0.04 and incongruent/right *M* = 1.46 ± 0.04 conditions).

As a final *lme* analysis, we better address the happiness advantage analysing the individual values of happiness advantage, with a *lme* model including face orientation, spatial congruency and target absolute emotional intensity as fixed factors, *r*_c_ = 0.89, 95% CI [0.87, 0.91]. As for Experiment 1, the *lme* model did not revealed significant interactions or main effects neither for upright, *r*_c_ = 0.75, 95% CI [0.70, 0.80], nor for inverted face orientations, *r*_c_ = 0.80, 95% CI [0.75, 0.84]. However, differently from Experiment 1, with the cross-range emotional pair of Experiment 2, we found a rather strong happiness advantage. This result is evidenced by contrasting individual values of happiness advantage against the reference null value standing for an unbiased motor reactivity (*M*_values of happiness advantage_ = 0.18 ± 0.03, vs. 0, *t*(45) = 5.22, *p* < 0.001, *d* = 1.56). All our experimental conditions indeed revealed a rather robust happiness advantage: fully emotional pairs (Fig. 4g, h), *M*_Fully emotional_ = 0.20 ± 0.04, vs. 0, *t*(45) = 5.50, *p* < 0.001, *d* = 1.64, displayed in both upright, *M*_Upright/Fully emotional_ = 0.18 ± 0.04, *t*(45) = 4.73, *p* < 0.001, *d* = 1.41, and inverted, *M*_Inverted/Fully emotional_ = 0.22 ± 0.04, *t*(45) = 5.93, *p* < 0.001, *d* = 1.77, orientation; as well as intermediate emotional pairs (Fig. 4e, f), *M*_Intermediate emotional_ = 0.16 ± 0.03, vs. 0, *t*(45) = 4.64, *p* < 0.001, *d* = 1.38, displayed in both upright, *M*_Upright/Intermediate emotional_ = 0.15 ± 0.04, *t*(45) = 4.21, *p* < 0.001, *d* = 1.26, and inverted, *M*_Inverted/Intermediate emotional_ = 0.17 ± 0.04, *t*(45) = 4.60, *p* < 0.001, *d* = 1.37, orientation.

### Discussion joining the results of Experiment 1 and 2

Results of Experiment 2 revealed a robust happiness advantage when the emotional comparison was performed on cross-range emotional pairs, different from Experiment 1, in which only half-range emotional pairs were used. This happiness advantage is independent of both face orientation and spatial congruency. The difference between Experiment 1 and 2 is likely due to the fact that, differently from Fantoni et al. (2019), in our two experiments, half- and cross-range emotional pairs were studied separately. Thus, to further support the conclusion that the typical pattern of responses rising from attentional capture in emotion comparison task involving ESC is independent from face orientation and can account for the joining of motor reactivity resulting from responses to cross-range and half-range emotional pairs in our two experiments, we ran a further *lme* analysis to replicate those performed by Fantoni et al. (2019). In particular, we joined the patterns of individual response speeds resulting from Experiment 1 and Experiment 2, including the variable Experiment (Experiment 1, Experiment 2) as an additional fixed factor together with average emotion intensity (− 50, 0, and + 50), face orientation (upright vs. inverted), response side (left vs. right) and spatial congruency (congruent vs. incongruent), and the random structure now including also the experiment, beyond the factors included in the *lme* analysis of Experiment 1. Notably, in such analysis the two conditions of target absolute emotion intensity of Experiment 2 were pooled as the average emotion intensity of cross-range emotional pairs was constrained to be null.

The key result was that the fixed structure of the best fitting *lme* model resulted to be simpler than the one including the full interaction between the whole set of experimental factors entered into the analysis with 31 *df*, *r*_c_ = 0.74, 95% CI [0.73, 0.74]. The best fitting *lme* model was one with 20 *df* and accounting for the exact same amount of variance of the full fixed structure *lme* model but optimizing the goodness of fit, *χ*^2^(11) = 5.25, *p* = 0.92, AIC(20) = 5826.1 vs. AIC(31) = 5842.8; BIC(20) = 5971.6, vs. BIC(31) = 6068.3. This model did not include the main effects of response side and spatial congruency, while including the following additive components:

1. the average emotion intensity × response side × spatial congruency component modelling the ESC, namely typical crossover effect rising from attentional capture in emotion comparison tasks, *F*(1, 227.98) = 292.82, *p* < 0.001, *η*_*p*_^2^ = 0.562, 95% CI [0.480, 0.626];
2. the average emotion intensity component, modelling the emotional size effect, *F*(1, 22.29) = 162.76, *p* < 0.001, *η*_*p*_^2^ = 0.879, 95% CI [0.749, 0.922];
3. the face orientation component, modelling the global orientation dependence of motor reactivity in emotion comparison due to the face inversion effect, *F*(1, 767.03) = 63.37, *p* < 0.001, *η*_*p*_^2^ = 0.073, 95% CI [0.041, 0.110];
4. the experiment × response side × spatial congruency component, modelling the dependence of the happiness advantage from the *type of pair* used in the two experiments (absent with half-range pairs as in as in Experiment 1 vs. present with cross-range pairs as in Experiment 2), *F*(1, 777.68) = 54.16, *p* < 0.001, *η*_*p*_^2^ = 0.064, 95% CI [0.036, 0.101];
5. the experiment component *F*(1, 22.54) = 11.19, *p* < 0.01, *η*_*p*_^2^ = 0.332, 95% CI [0.049, 0.0552], modelling an overall slowing down of responses in half-range rather than cross-range displays [*lme* estimated *M*_Experiment 1_ = 1.13 ± 0.03, vs. *M*_Experiment 2_ = 1.56 ± 0.06, *t*(85.14) = 8.45, *p* < 0.001, *d* = 1.83].

Finally, we compared individual values of happiness advantage in Experiment 1 and Experiment 2 for facial-expression pairs which were comparable in terms of emotional distance (i.e., removing from the dataset of Experiment 2 the responses to cross-range emotional pairs with target absolute emotional intensity = 100). Results on individual values of happiness advantage corroborated the strong vs. null happiness advantage observed in Experiment 2 vs. 1, with a main effect of experiment, *F*(1, 86) = 12.87, *p* < 0.001, *η*_*p*_^2^ = 0.130, 95% CI [0.026, 0.263], characterized by larger happiness advantage for cross-range emotional pairs of Experiment 2 compared to half-range emotional pairs of Experiment 1, 0.16 ± 0.03, vs. 0.02 ± 0.03, *t*(86) = 3.59, *p* < 0.001, *d* = 1.00. No other main effects or interactions were found included Face Orientation (all *p*s > .06), which further demonstrates that the happiness advantage in our Experiments was orientation independent.

## General discussion

Previous findings (Baldassi et al., 2021; Fantoni et al., 2019) showed that motor reactivity in emotion comparison task is stimulus driven. Such a pattern of motor reactivity depends on an attentional capture phenomenon, which is regulated by the perceptual salience of emotional stimuli, and it is fully independent from the congruency with any mental spatial representation of valence, as well as from the type of task (direct or indirect, Fantoni et al., 2019) and representational domain (symbolic or non-symbolic, Baldassi et al., 2021). However, as regards facial expressions of emotion it remained unclear whether these general patterns of dependencies involve part-based or holistic processing. Here, we tested the hypothesis that a holistic processing could drive participants’ attention towards a target emotional face by comparing their motor reactivity to emotional expressions of upright and inverted faces. Since face inversion disrupts the holistic processing of a face, compromising the recognition of its identity (Thompson, 1980), we expected and found a significant slowing down in the pattern of motor reactivity with inverted faces compared to upright faces. By contrast, the typical crossover effect elicited by attentional capture in emotion comparison tasks was independent on face orientation. Specifically, for both upright and inverted face conditions motor reactivities were consistent with the ESC pattern found by Fantoni et al. (2019), namely, the choice speed increased as the absolute emotion intensity of the target face grew larger together with the average emotion of the pair. Hence, our results indicate that the facial expression detection driven by attentional capture in our emotional comparison task relies on part-based rather than holistic face processing.

This is consistent with previous evidence that face inversion overall slows—but does not alter—expression recognition or effects related to it, like spatial attention (Williams et al., 2005; Lipp et al., 2009). According with the literature, the lack of face inversion effect on emotion recognition stems from the detection of low-level facial features related to emotions. For example, Savage and Lipp (2015) (but see also Calvo & Nummenmaa, 2008) suggested that the detection of emotional expressions is due to the perception of prominent single features of the face (e.g., the teeth). The authors demonstrated that focusing attention on parts of the target that make it discriminable from distractors not only facilitates the recognition of an emotional target compared to neutral expressions but increases the detection of an emotion when a holistic processing of the stimulus is not possible, as in the case of inverted faces. Moreover, the presence of conspicuous facial features—particularly in the mouth region—can make some emotional expressions visually more salient than others (Calvo & Nummenmaa, 2008; Pallett & Meng, 2015). This last interpretation, however, has received conflicting support from the literature. For example, Fox and Damjanovic (2006) found an advantage in detecting angry faces compared to happy faces only with upright faces, but this difference was abolished by face inversion. Importantly, Savage and Lipp (2015) showed that the superiority of angry over happy faces could be modulated by combining in different ways half-range and cross-range emotional pairs in a same-different visual task irrespective of face inversion, similar to our pattern of results. This shows that even though the detection of emotional expressions is based on the extrapolation of low-level facial features, this process can be more complex, being feedforward affected by contextual information. As consistent with this idea, our results show a more intricated scenario, as attentional capture in emotion comparison is affected by several factors including the absolute intensity of the target emotion, the average intensity of the emotional pair and the type of emotional pair being half-range or cross-range, regardless of face orientation.

The fact that attentional capture in the presence of half-range emotional pairs (Experiment 1) was independent from the face orientation further supports the finding of Fantoni et al. (2019) that this phenomenon involves the prioritization of highly emotional over intermediate emotional faces in early sensory processing (Öhman et al., 2001; Sabatinelli et al., 2005; Vuilleumier et al., 2003). Such a prioritization would be responsible for both the fast response to emotional target faces when displayed together with an intermediate flanker and the slow response to the intermediate target face when presented with an emotional flanker. Specifically, in the latter condition the emotional flanker face would irresistibly capture participants’ attention, interfering with the target detection (West et al., 2009). Notably, this effect was independent from the compatibility between the side of responses and any spatial mental representation of emotional valence hypothesized by several studies (e.g., Holmes & Lourenco, 2011; Holmes et al., 2019; Jansari et al., 2000). Our effect is rather consistent with the general idea that people might implicitly spatialize emotions accordingly with perceptually salient features of facial expressions (i.e., the mouth size being small for angry, medium for intermediate, and large for happy facial expressions as suggested by Pitt & Casasanto, 2018).

Furthermore, our finding is consistent with an orientation independent emotional size effect with globally faster responses for emotional pairs with positive rather than negative average emotion intensity displayed in upright and inverted orientation. However, differently from the results of Fantoni et al. (2019) in which half-range and cross-range emotional pairs were randomized within the same experimental block, in Experiment 1 we found no evidence for the happiness advantage using only half-range pairs. The happiness advantage was instead robust in Experiment 2 using cross-range emotional pairs in both upright and inverted orientations. The combination of results of Experiment 1 and 2 thus shows that the happiness advantage arises when the emotional intensity of the two faces within a pair is similar, although with opposite valence, as in cross-range emotional pairs, but not when it is different, as in half-range emotional pairs. This is consistent with attentional capture driven by perceptual salience, given that two faces with a similar emotional intensity have the same likelihood to capture attention, thus generating an attentional conflict that can be solved only on the basis of perceptual features. In particular, we hypothesized and found that, to solve this conflict, the attentional system prioritizes the emotional face that is perceptually more salient within a pair: the happiest, as expected with realistic faces used in the current experiments (Becker et al., 2011, 2012; Fantoni & Gerbino, 2014; Fantoni et al., 2016).

Notably, the main effect of Experiment revealed by the joint analysis of both Experiment 1 and 2 is in line with results by Bimler et al. (2013), supporting the idea that the inclusion of a neutral face in an emotional pair impair motor reactivity in emotion comparisons relative to the case in which both faces are fully emotional. This is consistent with the possibility that categorical perception may have played a role in shaping motor reactivity in our emotion comparison task (Cheetham et al., 2015; MacDorman & Chattopadhyay, 2016). We indeed consistently found that pair of faces with the same emotional distance of half-range emotional pairs but belonging to ostensibly different emotions as in the case of cross-range emotional pairs (with target absolute emotional intensity = 50) were chosen markedly faster of about 0.15 ± 0.05 unit of speeds, *t*(85.40) = 2.83, *p* < 0.01, *d* = 0.61.

## Conclusions

Relative to the original study of Fantoni et al. (2019), the present study further confirms the central role of attentional capture in emotion comparison, demonstrating for the first time its orientation independence. This independence is relevant for a full understanding of whether the type of perceptual processing impacts spatial attention in presence of emotionally salient stimuli. In particular, regardless of the type of perceptual processing, being either holistic, as likely occurring for faces displayed in upright orientation, or part-based, as likely occurring for faces displayed in inverted orientation (Piepers & Robbins, 2012; Valentine, 1988), similar crossover patterns of attentional capture in emotion comparison are observed. Attentional capture in emotion comparison is thus likely to be independent from the modalities in which human process facial expressions. Future investigations are necessary to further study the occurrence of the happiness advantage when inverted faces are randomized within stimuli including/not including the cut-off (i.e., the neutral) face of the emotional series. We also believe that our emotion comparison task merits further research to explore the extent to which the crossover pattern typical of responses rising from attentional capture in emotion comparison tasks might generalize to different pairs of emotional expressions (e.g., a fear *vs* a surprise face).

## Supplementary Information

Below is the link to the electronic supplementary material.

Supplementary file1 (XLSX 836 KB)

## Data Availability

All raw data generated in this study are included in this published article as supplemental materials file (Raw_data.xlsx).
